# Diagnose und Therapie der Osteoporose bei Patienten mit chronischer Niereninsuffizienz: Gemeinsame Leitlinie der Österreichischen Gesellschaft für Knochen- und Mineralstoffwechsel (ÖGKM), der Österreichischen Gesellschaft für Physikalische Medizin und Rehabilitation (ÖGPMR) und der Österreichischen Gesellschaft für Nephrologie (ÖGN)

**DOI:** 10.1007/s10354-022-00989-0

**Published:** 2022-12-21

**Authors:** Daniel Cejka, Robert Wakolbinger-Habel, Emanuel Zitt, Astrid Fahrleitner-Pammer, Karin Amrein, Hans Peter Dimai, Christian Muschitz

**Affiliations:** 1 Abteilung für Innere Medizin III, Nieren- und Hochdruckerkrankungen, Transplantationsmedizin, Rheumatologie, Akutgeriatrie, Ordensklinikum Linz – Krankenhaus der Elisabethinen, Fadingerstr. 1, 4020 Linz, Österreich; 2 Department of Physical and Rehabilitation Medicine (PRM), Vienna Healthcare Group – Clinic Donaustadt, Langobardenstr. 122, 1220 Wien, Österreich; 3 Department of Internal Medicine 3 (Nephrology and Dialysis), Feldkirch Academic Teaching Hospital, Feldkirch, Österreich; 4 Vorarlberg Institute for Vascular Investigation and Treatment (VIVIT), Feldkirch, Österreich; 5 Agency for Preventive and Social Medicine (aks), Bregenz, Österreich; 6 Division of Endocrinology and Diabetology, Medical University of Graz, Auenbruggerplatz 15, 8036 Graz, Österreich; 7 Medical Department II – VINFORCE, St. Vincent Hospital Vienna (Barmherzige Schwestern Krankenhaus Wien), Stumpergasse 13, 1060 Wien, Österreich

**Keywords:** Osteoporosis, Chronic kidney disease, Therapy, Fracture, FRAX, Osteoporose, Chronische Niereninsuffizienz, Therapie, Fraktur, FRAX

## Abstract

**Definition und Epidemiologie:**

- Chronische Niereninsuffizienz („chronic kidney disease“ [CKD]): Abnormität der Nierenstruktur oder Nierenfunktion für länger als 3 Monate. Stadieneinteilung der CKD anhand GFR und Albuminurie (not graded).
- Osteoporose: Erkrankung des Skeletts (verminderte Knochenmasse, Störung der Mikroarchitektur) mit erhöhtem Knochenbruchrisiko. Bei einem T‑Score ≤ −2,5 liegt definitionsgemäß eine Osteoporose vor. Bei Auftreten einer Fraktur nach inadäquatem Trauma liegt, unabhängig vom T‑Score, eine manifeste Osteoporose vor (not graded).
- Die Prävalenz von Osteoporose und osteoporotischen Frakturen sowie die CKD nehmen weltweit zu (not graded).

**Pathophysiologie, Diagnostik und Therapie der Chronic Kidney Disease – Mineral and Bone Disorder (CKD-MBD):**

- Definition des CKD-MBD-Syndroms: Störung des Kalzium‑, Phosphat‑, Vitamin-D- und Parathormon(PTH)-Haushalts sowie renale Osteodystrophie und vaskuläre Kalzifikation (not graded).
- Knochenstoffwechsel bei renaler Osteodystrophie: gesteigerter, normaler oder verminderter Knochenumbau möglich (not graded).
- Regelmäßige Laborkontrollen von Kalzium, Phosphat, alkalischer Phosphatase, PTH und 25-OH-Vitamin D mit Kontrollintervall je nach CKD-Stadium werden empfohlen (2C).
- Therapieziele bei CKD-MBD:
  - Hyperkalzämie vermeiden (1C)
  - Erhöhtes Phosphat in Richtung Normalbereich senken (2C)
  - PTH im Normbereich bis leicht erhöht halten (2D)
  - Vitamin-D-Mangel vermeiden bzw. beheben (1C)

**Diagnostik und Risikostratifizierung der Osteoporose bei CKD:**

- Densitometrie (mittels Dual Energy X‑ray Absorptiometry [DXA]): Niedriger T‑Score korreliert in allen Stadien der CKD mit höherem Frakturrisiko (not graded).
  - Verdopplung des Frakturrisikos pro Abnahme des T‑Scores um 1 Einheit (not graded).
  - T‑Score > −2,5 schließt eine Osteoporose nicht aus (not graded).
  - Falsch-hohe LWS-KMD-Messergebnisse können unter anderem bei aortaler Verkalkung, degenerativen Wirbelsäulenveränderungen (Osteophyten) oder bei bereits eingebrochenen Wirbelkörpern vorkommen (not graded).
- FRAX: Anwendung in allen CKD-Stadien orientierend möglich (1C).
- Knochenstoffwechselmarker: Bestimmung in Einzelfällen zum Therapiemonitoring (2D).
- Knochenbiopsie: In Einzelfällen, insbesondere bei CKD G5 (eGFR < 15 ml/min/1,73 m^2^) und CKD G5D (Dialyse) erwägen (2D).

**Spezifische Therapie der Osteoporose bei CKD:**

- Hypokalziämie vor Einleitung einer spezifischen Osteoporosetherapie ausgleichen (1C)
- Bei CKD G1–G2 (eGFR ≥ 60 ml/min/1,73 m^2^): Behandlung der Osteoporose wie für die Allgemeinbevölkerung empfohlen (1A).
- Bei CKD G3–G5D (eGFR < 60 ml/min/1,73 m^2^ bis Dialysestadium): primär Behandlung der laborchemischen Zeichen einer CKD-MBD (2C).
- Bei CKD G3 (eGFR 30–59 ml/min/1,73 m^2^) mit PTH im Normbereich und osteoporotischer Fraktur und/oder hohem Frakturrisiko gemäß FRAX: Behandlung der Osteoporose wie für die Allgemeinbevölkerung empfohlen (2B).
- Bei CKD G4–5 (eGFR < 30 ml/min/1,73 m^2^) und osteoporotischer Fraktur (Sekundärprävention): Osteoporosetherapie individualisiert empfohlen (2C).
- Bei CKD G4–5 (eGFR < 30 ml/min/1,73 m^2^) mit hohem Frakturrisiko (z. B. FRAX-score > 20 % für eine „major osteoporotic fracture“ oder > 5 % für eine Hüftfraktur) ohne osteoporotische Fraktur (Primärprävention): Osteoporosetherapie erwägen und ggf. auch einleiten (2D).
- Antiresorptive Behandlung bei CKD G4–5 (eGFR < 30 ml/min/1,73 m^2^): Kalziumkontrolle 1 bis 2 Wochen nach Therapiebeginn (1C).

**Physikalisch-rehabilitative Maßnahmen:**

- Krafttraining großer Muskelgruppen dreimal wöchentlich (1B).
- Ausdauertraining viermal wöchentlich 40 min (1B).
- Koordinationstraining/Balanceübungen dreimal wöchentlich (1B).
- Beweglichkeitsübungen drei- bis siebenmal wöchentlich (1B).

## Abstract (en)

**Definition and epidemiology:**

- Chronic kidney disease (CKD): abnormalities of kidney structure or function, present for over 3 months. Staging of CKD is based on GFR and albuminuria (not graded).
- Osteoporosis: compromised bone strength (low bone mass, disturbance of microarchitecture) predisposing to fracture. By definition, osteoporosis is diagnosed if the bone mineral density T‑score is ≤ −2.5. Furthermore, osteoporosis is diagnosed if a low-trauma (inadequate trauma) fracture occurs, irrespective of the measured T‑score (not graded).
- The prevalence of osteoporosis, osteoporotic fractures and CKD is increasing worldwide (not graded).

**Pathophysiology, diagnosis and treatment of chronic kidney disease-mineral and bone disorder (CKD-MBD):**

- Definition of CKD-MBD: a systemic disorder of mineral and bone metabolism due to CKD manifested by either one or a combination of the following: abnormalities of calcium, phosphorus, PTH, or vitamin D metabolism; renal osteodystrophy; vascular calcification (not graded).
- Increased, normal or decreased bone turnover can be found in renal osteodystrophy (not graded).
- Depending on CKD stage, routine monitoring of calcium, phosphorus, alkaline phosphatase, PTH and 25-OH-vitamin D is recommended (2C).
- Recommendations for treatment of CKD-MBD:
  - Avoid hypercalcemia (1C).
  - In cases of hyperphosphatemia, lower phosphorus towards normal range (2C).
  - Keep PTH within or slightly above normal range (2D).
  - Vitamin D deficiency should be avoided and treated when diagnosed (1C).

**Diagnosis and risk stratification of osteoporosis in CKD:**

- Densitometry (using dual X‑ray absorptiometry, DXA): low T‑score correlates with increased fracture risk across all stages of CKD (not graded).
  - A decrease of the T‑score by 1 unit approximately doubles the risk for osteoporotic fracture (not graded).
  - A T-score ≥ −2.5 does not exclude osteoporosis (not graded).
  - Bone mineral density of the lumbar spine measured by DXA can be increased and therefore should not be used for the diagnosis or monitoring of osteoporosis in the presence of aortic calcification, osteophytes or vertebral fracture (not graded).
- FRAX can be used to aid fracture risk estimation in all stages of CKD (1C).
- Bone turnover markers can be measured in individual cases to monitor treatment (2D).
- Bone biopsy may be considered in individual cases, especially in patients with CKD G5 (eGFR < 15 ml/min/1.73 m^2^) or CKD 5D (dialysis).

**Specific treatment of osteoporosis in patients with CKD:**

- Hypocalcemia should be treated and serum calcium normalized before initiating osteoporosis therapy (1C).
- CKD G1–G2 (eGFR ≥ 60 ml/min/1.73 m^2^): treat osteoporosis as recommended for the general population (1A).
- CKD G3–G5D (eGFR < 60 ml/min/1.73 m^2^ to dialysis): treat CKD-MBD first before initiating osteoporosis treatment (2C).
- CKD G3 (eGFR 30–59 ml/min/1.73 m^2^) with PTH within normal limits and osteoporotic fracture and/or high fracture risk according to FRAX: treat osteoporosis as recommended for the general population (2B).
- CKD G4–5 (eGFR < 30 ml/min/1.73 m^2^) with osteoporotic fracture (secondary prevention): Individualized treatment of osteoporosis is recommended (2C).
- CKD G4–5 (eGFR < 30 ml/min/1.73 m^2^) and high fracture risk (e.g. FRAX score > 20% for a major osteoporotic fracture or > 5% for hip fracture) but without prevalent osteoporotic fracture (primary prevention): treatment of osteoporosis may be considered and initiated individually (2D).
- CKD G4–5D (eGFR < 30 ml/min/1.73 m^2^ to dialysis): Calcium should be measured 1–2 weeks after initiation of antiresorptive therapy (1C).

**Physical medicine and rehabilitation:**

- Resistance training prioritizing major muscle groups thrice weekly (1B).
- Aerobic exercise training for 40 min four times per week (1B).
- Coordination and balance exercises thrice weekly (1B).
- Flexibility exercise 3–7 times per week (1B).

## Präambel

Aus Gründen der einfacheren Lesbarkeit wird in dieser Leitlinie durchgehend die maskuline Form verwendet, es sind aber ausdrücklich alle Geschlechter inkludiert.

Diese Leitlinie richtet sich primär an niedergelassene Ärzte, die Patienten mit Osteoporose und chronischer Niereninsuffizienz betreuen. Der Schwerpunkt der Leitlinie liegt daher auf der Behandlung von Patienten mit chronischer Niereninsuffizienz ohne Nierenersatztherapie (ohne Dialyse oder Nierentransplantation). Das Ziel der Leitlinie ist es, eine alltagstaugliche, praxisrelevante Anleitung zum Management dieser Patienten bereitzustellen.

## Nomenklatur der Empfehlungen

### Not graded

Eine Zuteilung von Evidenzlevel und Evidenzgrad ist nicht möglich oder sinnvoll, wie etwa bei Begriffsdefinitionen

### Empfehlungslevel

Level 1: Empfehlung. Die meisten Patienten sollten so behandelt werden.

Level 2: Vorschlag. Individualisiertes Vorgehen für Patienten.

Level 3: Nicht empfohlen. Von dieser Behandlung wird abgeraten.

### Evidenzgrad

Grad A: hoch. Zum Beispiel mehrere randomisierte, kontrollierte Phase-III-Studien verfügbar

Grad B: mittelgradig. Zum Beispiel einzelne kontrollierte Phase-III-Studie oder mehrere prospektive Interventionsstudien verfügbar

Grad C: niedrig – z. B. Korrelationsstudien, Registerdaten verfügbar

Grad D: sehr niedrig – z. B. Fallserien, pathophysiologische Überlegungen, Expertenmeinung

## Definitionen und Epidemiologie

### Definition der chronischen Niereninsuffizienz – „chronic kidney disease“ (CKD)

Die CKD ist definiert als eine Abnormität der Nierenstruktur oder Nierenfunktion, die für länger als 3 Monate besteht [1]. Die Stadieneinteilung der CKD erfolgt anhand der exkretorischen Nierenfunktion (geschätzte glomeruläre Filtrationsrate [eGFR]) sowie Albuminurie (Tab. 1).

**Table 1:** 

**GFR-Kategorie**		
*G1*	≥ 90	ml/min/1,73 m^2^
*G2*	60–89	
*G3a*	45–59	
*G3b*	30–44	
*G4*	15–29	
*G5*	< 15	
**Albuminurie-Kategorie (UACR)**		
*A1*	< 30	mg/g
*A2*	30–300	
*A3*	> 300	

*GFR* glomeruläre Filtrationsrate, *UACR* Urin Albumin/Kreatinin-Ratio

### Definition der Osteoporose

Osteoporose ist eine Erkrankung des Skeletts, welche durch eine verminderte Knochenmasse sowie durch Störungen der Knochenmikroarchitektur zu einem erhöhten Knochenbruchrisiko führt [2]. Die am weitesten verbreitete Methode zur Bestimmung der Knochenmineraldichte (KMD) ist die Dual Energy X‑ray Absorptiometry (DXA), deren Ergebnis als T‑Score (dimensionslos) ausgedrückt wird. Der T‑Score ist die geschlechtsspezifisch angegebene Standardabweichung der gemessenen KMD vom Normwert gesunder, junger Erwachsener. Bei einem T‑Score von −2,5 oder darunter liegt laut Weltgesundheitsorganisation (WHO) definitionsgemäß eine Osteoporose vor. Zu beachten ist jedoch, dass etwa die Hälfte aller osteoporotischen Frakturen (Knochenbrüche ohne adäquates mechanisches Trauma) bei Patienten mit einem T‑Score > −2,5 auftreten [3, 4]. Auch bei Patienten mit einem T‑Score > −2,5 kann daher eine Osteoporose vorliegen. Bei Patienten mit Fraktur nach inadäquatem Trauma liegt unabhängig vom T‑Score eine manifeste Osteoporose vor.

### Epidemiologie der CKD und Osteoporose

Aufgrund der steigenden Lebenserwartung nimmt die Prävalenz chronischer Erkrankungen in vielen Ländern der sog. westlichen Welt stetig zu.

Die CKD (alle Stadien) betrifft knapp 10 % der Weltbevölkerung mit steigender Prävalenz [5]. Die Prävalenz von CKD ist in Österreich nicht genau bekannt, die Dunkelziffer liegt vermutlich hoch. Aktuelle Schätzungen gehen davon aus, dass zwischen 200.000 und 900.000 Menschen in Österreich von einer CKD betroffen sind [6].

Osteoporose ist ebenfalls häufig: Jedes Jahr treten weltweit etwa 9 Mio. osteoporotische Frakturen auf, davon sind ca. 1,6 Mio. hüftnahe Frakturen [7]. In Österreich kommt es zu etwa 92.000 osteoporotischen Frakturen pro Jahr, etwa 16.000 davon sind hüftnahe Frakturen [8]. In Österreich ist die altersstandardisierte Hüftfrakturinzidenz seit etwa 2005 erfreulicherweise rückläufig, aufgrund der demografischen Entwicklung mit zunehmendem Durchschnittsalter der Bevölkerung ist die Prävalenz der Hüftfrakturen in den letzten 10 Jahren jedoch unverändert hoch [9].

Eine häufige Komplikation der chronischen Niereninsuffizienz ist die Störung des Mineral- und Knochenstoffwechsels, genannt „chronic kidney disease – mineral and bone disorder“ (CKD-MBD) [10], sodass die Diagnostik und Therapie der Osteoporose bei Patienten mit chronischer Niereninsuffizienz an diese pathophysiologischen Vorgänge angepasst werden sollte.

## Pathophysiologie, Diagnostik und Therapie der Chronic Kidney Disease – Mineral and Bone Disorder (CKD-MBD)

**Figure 1:**
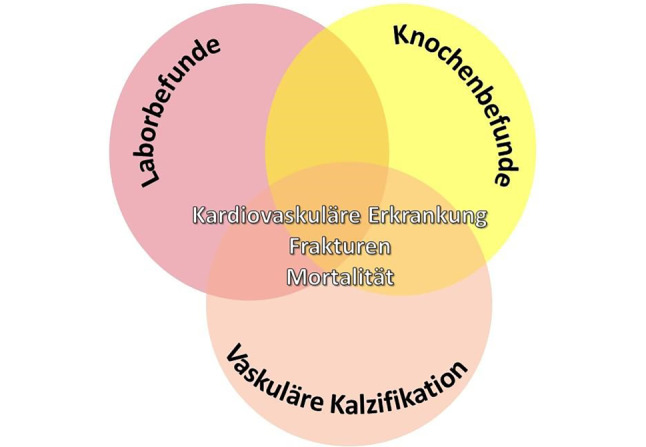

Die Regulation des Knochen- und Mineralstoffwechsels hängt entscheidend von der Kontrolle der Kalzium- und Phosphathomöostase ab. Die Hormone Parathormon (PTH), Calcitriol (1,25(OH)2D3), Calcidiol (25(OH)D3) und FGF23 regulieren den Kalzium- und Phosphathaushalt und beeinflussen Darm, Nebenschilddrüsen, Knochen und Nieren. Die Nieren spielen dabei eine wichtige Rolle, weil sie bei mehreren dieser Mechanismen als Zielorgan die Feinregulation übernehmen, um die Kalzium- und Phosphathomöostase aufrechtzuerhalten. Daraus ergibt sich, dass Nierenfunktionsstörungen zu gravierenden Veränderungen im Mineral- und Knochenstoffwechsel führen [11]. Das klinische Syndrom, welches die biochemischen Veränderungen des Mineralstoffwechsels (Hypokalzämie, Hyperphosphatämie, Hyperparathyreoidismus, Calcitriolmangel, erhöhte FGF23-Konzentration), die histologischen Knochenveränderungen (zusammenfassend als „renale Osteodystrophie“ bezeichnet), Knochenfrakturen sowie vaskuläre und valvuläre Verkalkungen umfasst, wird unter dem Begriff „CKD-MBD“ (Chronic Kidney Disease – Mineral and Bone Disorder) zusammengefasst (Abb. 1, [10]). Insbesondere vaskuläre [12, 13] und valvuläre [14] Kalzifizierung sind direkt mit einem gesteigerten Risiko für kardiovaskuläre Morbidität und Mortalität sowie einer exzessiv erhöhten Gesamtmortalität assoziiert [15–18].

### Pathophysiologie

In der Pathogenese der CKD-MBD kommt es mit abnehmender Nierenfunktion adaptiv als früheste Veränderung zu einer Erhöhung der FGF23(„fibroblast growth factor 23“)-Konzentration und zur Abnahme der funktionellen renalen Masse. Daraus resultiert eine reduzierte Synthese von Calcitriol (hormonell aktives Vitamin D), wodurch die intestinale Kalziumaufnahme abnimmt und die Serumkalziumkonzentration abfallen würde. Dies wiederum stimuliert die Synthese und Freisetzung von PTH. Der resultierende Hyperparathyreoidismus bewirkt einen erhöhten Knochenumsatz und steigert die Knochenresorption, stimuliert gleichzeitig auch die renale Calcitriolbildung. Diese Mechanismen zielen auf eine Normalisierung der Serumkalziumkonzentration ab. Erhöhtes FGF23 (vorwiegend von Osteozyten und Osteoblasten sezerniert) und PTH fördern die renale Phosphatexkretion, wodurch sowohl für Kalzium als auch für Phosphat normale Serumkonzentrationen in der Regel bis in das CKD-Stadium 4 aufrechterhalten werden können [19, 20]. Trotz synergistischer Effekte von PTH und FGF23 auf die renale Phosphatexkretion unterscheiden sie sich durch ihre gegenteilige Wirkung auf die Calcitriolsynthese: FGF23 inhibiert, PTH stimuliert die Calcitriolsynthese.

**Figure 2:**
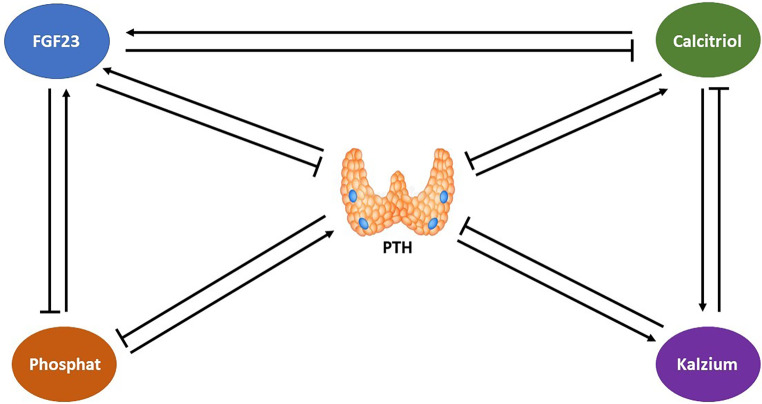

Kalzium und Calcitriol üben ihre Wirkung auf die Nebenschilddrüse über spezifische Rezeptoren aus: den membranständigen Ca-sensing-Rezeptor (CaSR) und den als Transkriptionsfaktor wirkenden nukleären Vitamin-D-Rezeptor (VDR). Bei Hypokalzämie kommt es CaSR-vermittelt innerhalb von Sekunden bis Minuten zur Freisetzung von gespeichertem PTH, im Fall erhöhter Kalziumwerte sinkt die PTH-Sekretion. Bei länger bestehender niedriger Kalziumkonzentration wird die PTH-Synthese gesteigert, eine erhöhte Kalziumkonzentration führt zur Reduktion der PTH-Synthese [21]. Der CaSR ist auch der Phosphatsensor der Nebenschilddrüsenzelle. Bei Hyperphosphatämie wird die Aktivität des CaSR gehemmt, wodurch sich die phosphatinduzierte gesteigerte PTH-Sekretion erklären lässt [22]. Im Gegensatz zu Kalzium und Phosphat bindet Calcitriol an den VDR, der dessen Wirkung über die verminderte Transkription des *PTH*-Gens und eine reduzierte PTH-Synthese vermittelt. In den CKD-Stadien 4 und 5 gelingt keine adäquate Kontrolle mehr. Trotz gesteigerter FGF23- und PTH-Sekretion kommt es zu erhöhter Serumphosphatkonzentration und signifikant vermindertem Calcitriolspiegel. Bei fortgeschrittener CKD-MBD resultiert aus der Kalzium- und Calcitriolreduktion sowie der Hyperphosphatämie eine Nebenschilddrüsenhyperplasie. In weiterer Folge entwickelt sich ein renaler sekundärer Hyperparathyreoidismus (sHPT) [23, 24]. Die Abb. 2 fasst die Beziehungen der entscheidenden Regulationsparameter Kalzium, Phosphat, Calcitriol, FGF23 und PTH zusammen.

### Renale Osteodystrophie

Aufgrund der geschilderten pathophysiologischen Vorgänge würde bei Patienten mit fortgeschrittener CKD eine hohe Prävalenz an sHPT mit damit einhergehend gesteigertem Knochenumbau erwartet werden. Tatsächlich jedoch hat sich das histomorphometrische Spektrum in den letzten Jahrzehnten gewandelt, und ein verminderter Knochenumsatz ist zur vorherrschenden Knochenmanifestation im Rahmen der CKD-MBD geworden. Die alternde CKD-Population, Zunahme der Diabetesprävalenz und exzessive Kalzium- und Calcitriolsubstitution werden ursächlich vermutet [25–27]. Die Kenntnis der unterschiedlichen Ausprägungen der renalen Osteodystrophie (erhöhter oder erniedrigter Knochenstoffwechsel) ist auch bezüglich möglicher Therapien relevant.

Bei Patienten mit CKD resultiert aus den Veränderungen des Knochenumbaus ein fragilerer Knochen mit erhöhter Brüchigkeit. Der Knochenumbau betrifft sowohl den trabekulären als auch den kortikalen Knochen. Beide nehmen bei CKD-Patienten ab, wobei aufgrund des stärkeren Effekts von PTH auf den kortikalen Knochen dessen Verlust im Vordergrund steht. Es resultiert eine dünne, poröse, eher „trabekulierte“ Kortikalis (Ersatz der inneren Kortikalisschicht durch morphologisch trabekulären Knochen), welche insbesondere in den langen Röhrenknochen die Knochenbrüchigkeit stark erhöht. Bei ausgeprägtem Mangel an Vitamin D kann es auch zu einer Mineralisationsstörung (Osteomalazie) kommen.

### Labordiagnostik

Die Routinelabordiagnostik der CKD-MBD umfasst die Bestimmung der Serumkonzentrationen von Kalzium, Phosphat, Calcidiol [25(OH)D], PTH und alkalischer Phosphatase neben den zur CKD-Diagnostik üblichen Serum- und Harnparametern. Zu beachten ist, dass die alkalische Phosphatase nur bei Patienten ohne Lebererkrankung für die CKD-MBD-Diagnostik verwertbar ist. Eine Alternative ist hier die Bestimmung der knochenspezifischen alkalischen Phosphatase. Die Bestimmung von FGF-23 ist zwar pathophysiologisch interessant, hat aber noch keinen Einzug in die Routinediagnostik gefunden. Die Kidney Disease Improving Global Outcomes(KDIGO)-CKD-MBD-Leitlinien empfehlen ab dem CKD-Stadium G3 die regelmäßige Kontrolle dieser Parameter [10], wobei sich die Untersuchungsintervalle mit zunehmender Nierenfunktionseinschränkung verkürzen (Tab. 2).

**Table 2:** 

	Kalzium	Phosphat	PTH	AP	Calcidiol [25(OH)D]
G3	6–12 Monate	6–12 Monate	6–12 Monate	Einmalig	Jährlich^a^
G4	3–6 Monate	3–6 Monate	6–12 Monate	6–12 Monate	Jährlich^a^
G5	1–3 Monate	1–3 Monate	3–6 Monate	6–12 Monate	Jährlich^a^

*PTH* Parathormon, *AP* alkalische Phosphatase

^a^Wiederholt je nach Ausgangswert und Intervention (Supplementation)

Grundsätzlich wird empfohlen, dass Therapieentscheidungen auf Trends von Laborparametern und nicht auf Einzelwerten beruhen sollen und die Gesamtheit der vorliegenden Befunde zu Störungen des Kochen- und Mineralstoffwechsels gemeinsam berücksichtigt werden soll [10]. Die Tab. 3 fasst die aktuell gültigen Zielbereiche für die Laborparameter zusammen.

**Table 3:** 

	CKD G3–G5	Dialyse (CKD 5D)
Phosphat	Erhöhtes Phosphat in Richtung Normalbereich senken	Erhöhtes Phosphat in Richtung Normalbereich senken
Kalzium	Hyperkalzämie vermeiden	Hyperkalzämie vermeiden
PTH	Für CKD G4 und G5 im Normbereich bis leicht erhöht	Zwischen dem 2‑ und dem 9fachen des oberen Normbereichs des jeweiligen verwendeten PTH-Assays
Calcidiol [25(OH)D]	Vermeiden eines Vitamin-D-Mangels Ziel-25(OH)D 20–40 ng/ml	Vermeiden eines Vitamin-D-Mangels Ziel-25(OH)D 20–40 ng/ml

*CKD* „chronic kidney disease“, *PTH* Parathormon

### Therapie der CKD-MBD

Bislang ist nicht gesichert, ob eine Verbesserung der biochemischen Parameter auch zu einem verbesserten Patienten-Outcome führt. Die Therapieempfehlungen sind daher aktuell als Best-practice-Empfehlungen anzusehen. Um die oben genannten laborchemischen Ziele (Tab. 3) zu erreichen, können folgende Interventionen angewendet werden:

#### Phosphatrestriktion und Phosphatbinder

Der exakte Schwellenwert, dessen Überschreiten zu einem signifikant erhöhten kardiovaskulären Risiko führt, ist nicht definitiv bekannt und variiert in den Beobachtungsstudien zwischen 1,6 und 2,3 mmol/l (e. g. [15]). Die internationalen KDIGO-Leitlinien empfehlen, bei Patienten mit eingeschränkter Nierenfunktion und Hyperphosphatämie die Serumphosphatwerte „in Richtung Normbereich“ zu senken, ohne dass definierte Zielkonzentrationen angegeben werden [10].

Die Therapie der Hyperphosphatämie umfasst im Wesentlichen eine diätetische Phosphatrestriktion und die Gabe von Phosphatbindern, bei Dialysepatienten zusätzlich die Phosphatelimination mittels effizienter Dialyse.

Eine diätetische Phosphatrestriktion ist zwangsläufig auch mit einer Einschränkung der oralen Eiweißzufuhr verbunden, da eiweißreiche Nahrung die Hauptquelle der diätetischen Phosphatzufuhr darstellt [28]. Eine reduzierte Eiweißzufuhr kann bei fortgeschrittener CKD aber zu Malnutrition führen, was wiederum mit einer erhöhten Mortalität vergesellschaftet ist [29, 30]. Eine Möglichkeit der proteinunabhängigen Phosphateinschränkung besteht darin, Fertiggerichte und Getränke mit phosphathaltigen Zutaten zu vermeiden [31, 32]. Im Gegensatz zu den natürlichen Phosphatquellen wie Fleisch und Milchprodukte erfolgt hierbei eine alleinige Phosphatzufuhr ohne energiereichen Eiweißanteil. Eine weitere Option besteht darin, Eiweißquellen mit niedrigem Phosphat/Protein-Verhältnis (v. a. pflanzliche Eiweiße, z. B. Hülsenfrüchte) zu bevorzugen [33].

Orale Phosphatbinder vermindern die intestinale Phosphatresorption und können so die Serumphosphatwerte senken. Bei Patienten im CKD-Stadium G3–5 kann durch eine Phosphatbindertherapie die Serumphosphatkonzentration wenig bis gar nicht gesenkt werden (e. g. [34]). Bei Patienten im CKD-Stadium 3–4 konnte bislang auch kein Vorteil einer Phosphatbindertherapie bezüglich kardiovaskulärer Surrogatendpunkte (z. B. Progression von Gefäßverkalkung oder Gefäßsteifigkeit) gezeigt werden [35, 36]. Eine Phosphatbindertherapie ist daher im CKD-Stadium 3 und 4 nicht indiziert. Bei Patienten im CKD-Stadium 5 mit deutlich erhöhten Serumphosphatwerten bzw. bei Dialysepatienten (CKD 5D) ist in den meisten Fällen eine Phosphatbindertherapie notwendig und effektiv, um die Serumphosphatspiegel im aktuell empfohlenen Bereich zu halten. Auch bei Dialysepatienten fehlt jedoch der definitive Wirksamkeitsnachweis einer Phosphatbindertherapie bezüglich Senkung von kardiovaskulären Ereignissen oder Mortalität.

Aktuell verfügbare Phosphatbinder sind: Kalziumkarbonat, Kalziumazetat, Aluminiumhydroxid, Sevelamerkarbonat, Lanthankarbonat, Magnesium‑/Kalziumkarbonat, Magnesiumkarbonat/Kalziumazetat, Eisenzitrat und Sucroferric Oxyhydroxid. Derzeit kann keiner der verfügbaren Phosphatbinder per se favorisiert werden, weil keine Daten existieren, die eine bewiesene Überlegenheit eines Phosphatbinders oder einer Phosphatbinderklasse hinsichtlich relevanter klinischer Parameter belegen.

#### Vitamin-D-Therapie

Ein Vitamin-D-Mangel [25(OH)D < 30 ng/ml] stellt bei Patienten mit moderat bis schwer eingeschränkter Nierenfunktion (CKD-Stadium G3–4) eine mögliche Ursache eines sekundären Hyperparathyreoidismus (sHPT) dar, sodass eine Spiegelbestimmung und ggf. Substitution (meist mit Vitamin D_3_/Cholecalciferol) in dieser Konstellation empfohlen werden [10]. Während in frühen Stadien einer CKD ein sHPT erfolgreich mit nativem Vitamin D therapiert werden kann, ist der Nutzen einer solchen Substitution für den Knochen- und Mineralstoffwechsel in sehr späten Stadien inklusive Dialysepflichtigkeit (CKD G5 und G5D) begrenzt. Der Erfolg einer Supplementierung mit nativem Vitamin D im Hinblick auf die Erhöhung der 25(OH)D-Konzentration und eine damit einhergehende PTH-Senkung wird in einer großen rezenten Metaanalyse bisheriger randomisiert kontrollierter Studien bestätigt [37]. Eine ausreichende Dosierung und adäquate Therapiedauer sind Voraussetzung für das Erreichen und Aufrechterhalten eines suffizienten Vitamin-D-Spiegels [38]. Zur Beeinflussung des sHPT dürfte bei CKD-Patienten eine höhere 25(OH)D-Zielkonzentration als > 20 ng/ml wie in der Allgemeinbevölkerung anzustreben sein, vermutlich im Bereich von 40–50 ng/ml [39–41]. Aktuell existiert für CKD-Patienten keine spezielle Dosierungsempfehlung. Die Expertenmeinung dieser Arbeitsgruppe ist daher, bei Nachweis einer Vitamin-D-Insuffizienz täglich 3000–4000 IE (oder wöchentlich 20.000–30.000 IE, je nach verfügbarem Präparat) zu verabreichen mit Kontrolle von 25(OH)D, PTH, Kalzium und Phosphat nach 8 bis 12 Wochen. Bei Erreichen einer suffizienten 25(OH)D-Konzentration empfehlen wir, in halber Dosierung fortzufahren. Falls dies bezüglich des Vitamin-D-Spiegels unzureichend sein sollte, empfehlen wir, die Initialdosis fortzuführen oder bei gesicherter Adhärenz unter regelmäßiger Laborkontrolle zu erhöhen [38].

Die Behandlung des sHPT mit Calcitriol (1,25-OH_2_-D) oder Vitamin-D-Analoga (Paricalcitol, Alfacalcidol) im Prädialysestadium (CKD G3-G5) sollte erst bei progredientem oder schwerem sHPT erfolgen [10]. Dies betrifft in der Praxis v. a. das Stadium CKD 5. Bei Dialysepatienten (Stadium G5D) mit Notwendigkeit zur PTH-senkenden Therapie stellen Calcitriol oder Vitamin-D-Analoga hingegen als Monotherapie oder in Kombination mit Kalzimimetika eine empfohlene Therapieoption dar [10]. Für keine der verfügbaren Substanzen ist eine gesicherte Überlegenheit hinsichtlich relevanter klinischer Parameter belegt, sodass derzeit die Substanzwahl individualisiert und kostensparend erfolgen kann.

#### Kalzimimetika

Kalzimimetika sind allosterische Modulatoren des CaSR, die die Empfindlichkeit des Rezeptors gegenüber extrazellulärem Kalzium erhöhen. Daraus resultiert eine verminderte PTH-Sekretion und Hemmung der NSD-Zellproliferation [42–44]. Mit der Reduktion der PTH-Spiegel geht auch eine Senkung der Serumkalzium‑, Serumphosphat- und FGF-23-Konzentration einher [45–47]. Derzeit stehen in Österreich zur sHPT-Therapie bei CKD Patienten im Stadium G5D 2 Kalzimimetika zur Verfügung: das täglich oral verabreichte Erstgenerationskalzimimetikum Cinacalcet sowie das parenteral am Ende jeder Hämodialyse applizierte Etelcalcetid als Vertreter der zweiten Generation. Mit beiden Kalzimimetika erreichen mehr Patienten die angestrebten biochemischen Zielwerte, eine anhaltende Kontrolle des sHPT über zumindest 3 Jahre ist für Cinacalcet dokumentiert [48]. Aufgrund der hypokalzämischen Wirkung ist eine regelmäßige Kontrolle der Serumkalziumspiegel notwendig; bei niedrigen Kalziumwerten entsprechend eine Dosisanpassung oder Kombination mit niedrig dosiertem Calcitriol bzw. Vitamin-D-Analogon indiziert. Bei Dialysepatienten besteht auch die Möglichkeit, die Kalziumkonzentration im Dialysat zu erhöhen.

Kalzimimetika sind für Patienten im Stadium CKD 5D (Dialyse), nicht jedoch für Patienten im Stadium CKD 3–5 zugelassen. Bei Nicht-Dialysepatienten sollten Kalzimimetika nur in begründeten Einzelfällen wie etwa bei persistierendem hyperkalzämischem, hypo- bis normophosphatämischem Hyperparathyreoidismus nach erfolgreicher Nierentransplantation verwendet werden („off-label“).

#### Parathyreoidektomie

Ein medikamentös therapierefraktärer sHPT mit PTH-Werten persistierend > 800–1000 pg/ml und gleichzeitig bestehender Hyperkalzämie oder Hyperphosphatämie, oder eine Kalziphylaxie mit gleichzeitigem HPT und fehlendem Ansprechen auf Kalzimimetika stellt eine Indikation zur Parathyreoidektomie dar [10, 49], dies betrifft v. a. Dialysepatienten. Durch die Einführung der Kalzimimetika hat die Parathyreoidektomie aber in der Therapie des sHPT stark an Bedeutung verloren. Die fehlende Möglichkeit einer „Feinregulierung“ der PTH-Senkung, die Endgültigkeit des Eingriffs und die oftmals notwendige höher dosierte Kalziumsupplementierung und Vitamin-D-Gabe mit konsekutiv erhöhtem Kalzifizierungsrisiko sind Nachteile dieses operativen Eingriffs.

## Diagnostik und Risikostratifizierung der Osteoporose bei CKD

### Densitometrie (DXA)

Die Messung der Knochenmineraldichte (KMD) mittels Densitometrie (Dual Energy X‑ray Absorptiometry [DXA]) ist die am weitesten verbreitete apparative Methode zur Abschätzung des Knochenbruchrisikos. Die DXA erfolgt meist an der Lendenwirbelsäule (LWS), der Hüfte („hip“) und der Oberschenkelhalsregion („femoral neck“ [FN]). DXA-Messungen des Radius (R) können v. a. bei stark fortgeschrittenen Stadien der Niereninsuffizienz (CKD 4–5) eine gewisse Mehraussage bringen, jedoch ist für die Knochendichte des Radius bezüglich Frakturprädiktion die Datenlage gering [50]. Ausgegeben werden die Ergebnisse der DXA entweder als T‑Score (dimensionslos, Standardabweichung vom Mittelwert der „peak bone mass“ von jungen Erwachsenen) oder in Gramm pro Quadratzentimeter (g/cm^2^). Definitionsgemäß und unabhängig vom CKD-Stadium besteht ab einem T‑Score < −1,5 eine Osteopenie, ab einem T‑Score < −2,5 eine Osteoporose. Eine niedrige Knochendichte ist ein unabhängiger Prädiktor für das Auftreten von Frakturen sowohl in der Allgemeinbevölkerung (e. g. [51]) als auch in allen Stadien der CKD inklusive des dialysepflichtigen Nierenversagens [52–56]. Zur Orientierung kann vereinfacht gesagt werden, dass sich pro Abnahme des T‑Scores um 1 Einheit die Frakturwahrscheinlichkeit in etwa verdoppelt [52, 56]. Patienten mit CKD weisen häufig degenerative Prozesse der LWS (z. B. Osteophyten) und auch häufig eine Verkalkung der abdominalen Aorta auf, beides Einflussfaktoren, die zu einer falsch hohen LWS-KMD-Messung führen können [57, 58]. Eine normale KMD in der LWS schließt daher bei CKD-Patienten eine Osteoporose nicht aus. Fällt die Knochendichte der LWS bei CKD-Patienten jedoch niedrig aus, kann von einer verminderten Knochenfestigkeit ausgegangen werden.

### Risikorechner – FRAX

Das Frakturrisiko hängt nicht nur von der KMD ab, sondern wird auch von einer Vielzahl weiterer Faktoren (z. B. Alter oder auch Nierenfunktion) beeinflusst. So hat nur ungefähr die Hälfte aller Patienten, die eine osteoporotische Fraktur erleiden, eine Knochendichte im definitionsgemäß osteoporotischen Bereich (T-Score < −2,5) [59]. Daher wurden verschiedene Frakturrisikorechner entwickelt, um das individuelle Risiko des Patienten besser abzuschätzen. Das „Fracture Risk Assessment Tool“ (FRAX) ist der weltweit am häufigsten verwendete Frakturrisikorechner. Ein großer Vorteil von FRAX ist, dass für Österreich länderspezifische Frakturdaten eingespeist wurden und FRAX somit eine gute Abschätzung des Frakturrisikos für österreichische Patienten ermöglicht (https://www.sheffield.ac.uk/FRAX/tool.aspx?country=16). Eine Limitation von FRAX ist, dass eine eingeschränkte Nierenfunktion nicht als Risikofaktor im FRAX eingegeben werden kann. Aufgrund der Datenlage [55, 60–62] kann FRAX auch bei CKD-Patienten empfohlen werden (Ausnahme: CKD im Dialysestadium). In den CKD-Stadien 4 und 5 wird jedoch das Frakturrisiko von FRAX systematisch unterschätzt (unabhängig ob im FRAX die FN-KMD eingegeben wird oder nicht). Insbesondere das Risiko für eine „major osteoporotic fracture“ (klinisch apparente Wirbelkörperfrakturen sowie Frakturen von Femur, Humerus oder Unterarm) wird durch FRAX bei CKD 4 und 5 unterschätzt. Je höher das CKD-Stadium, desto ausgeprägter die Unterschätzung. Das Risiko für eine Oberschenkelhalsfraktur (isoliert betrachtet) wird von FRAX mit zunehmendem CKD-Stadium etwas weniger ausgeprägt unterschätzt [61].

### Knochenstoffwechselparameter

In der klinischen Routine stehen verschiedene Knochenstoffwechselparameter zur Verfügung. Marker der Osteoblastenaktivität sind unter anderem: knochenspezifische alkalische Phosphatase („bone-specific alkaline phosphatase“ [BAP]), Osteocalcin (Oc) und Prokollagen Typ1 aminoterminales Propeptid (P1NP). Marker der Osteoklastenaktivität sind unter anderem: Carboxy-terminale Kollagen-Crosslinks (CTx, auch genannt Crosslaps) oder die Tartrat-resistente saure Phosphatase 5b („tartrate-resistant acidic phosphatase 5b“ [TRAP 5b]). Bei CKD erscheint als Osteoblastenmarker die BAP, als Osteoklastenmarker die TRAP 5b Vorteile zu haben, da diese im Gegensatz zu den kollagenbasierten Markern kaum renal eliminiert werden [63].

#### Frakturprädiktion

In der Allgemeinbevölkerung sind die Daten widersprüchlich, ob eine Bestimmung von Knochenstoffwechselparametern eine valide Frakturprädiktion erlaubt [64, 65]. Die Mehrzahl der Daten zur Frakturprädiktion mittels Knochenstoffwechselparametern bei CKD-Patienten wurde bei Dialysepatienten erhoben, mit uneinheitlichen Ergebnissen [66–69]. Bei Patienten im CKD-Stadium 4 und 5 (Nicht-Dialyse) ist die Datenlage sehr limitiert und erbrachte bislang keinen klaren Mehrwert gegenüber der Risikoabschätzung mittels DXA [70]. Insgesamt wird eine Bestimmung der Knochenstoffwechselparameter zur Abschätzung des Frakturrisikos generell nicht empfohlen.

#### Therapiewahl und Therapiemonitoring

Für die Allgemeinbevölkerung ist die Datenlage, ob eine Bestimmung der Knochenstoffwechselparameter vor Therapiebeginn die Effektivität einer Osteoporosebehandlung vorhersagen kann, uneinheitlich. Beispielsweise wäre es basierend auf pathophysiologischen Überlegungen naheliegend, dass Patienten mit deutlich erhöhtem Knochenstoffwechsel (und damit einhergehender überwiegender Knochenresorption) besonders von einer antiresorptiven Therapie profitieren sollten. In Post-hoc-Analysen konnte diese Hypothese zwar für Alendronat bestätigt werden [71], für Risedronat jedoch nicht [72]: Hier war die langfristige Reduktion der Frakturrate unabhängig von der Höhe der initial gemessenen Stoffwechselmarker. Für die Allgemeinbevölkerung wird daher die Bestimmung der Knochenstoffwechselparameter als Basis für die Therapiewahl nicht empfohlen, kann aber in Einzelfällen zum Monitoring einer medikamentösen Behandlung (insbesondere der Therapieadhärenz) verwendet werden [66]. Für Patienten mit CKD 4–5 liegen aktuell keine Daten zu Knochenstoffwechselparametern zur Vorhersage der Effektivität einer Osteoporosetherapie vor. Eine Bestimmung von Knochenstoffwechselparametern bei CKD-4- bis -5-Patienten kann im Einzelfall eine Hilfestellung zur Planung einer auf pathophysiologischen Überlegungen basierten rationalen Therapie sein (osteoanabol vs. antiresorptiv), die aber aktuell nicht evidenzbasiert ist.

### Knochenbiopsie

Nach wie vor ist die Beckenkammbiopsie der Goldstandard zur genauen Diagnostik der renalen Osteodystrophie [73], insbesondere des aktuell bestehenden Knochenstoffwechsels (erhöht/normal/erniedrigt), da die Knochenstoffwechselparameter bei fortgeschrittener Niereninsuffizienz nur eine geringe Aussagekraft aufweisen (s. oben). Da Knochenbiopsien invasiv und aufwendig in der Probenaufarbeitung sind (nicht-entkalkte Mikroschnitte mit anschließender Histomorphometrie), ist die klinische Verfügbarkeit gering und in Österreich auf wenige hoch spezialisierte Zentren beschränkt. Bei unklarer Befundkonstellation, wie sie insbesondere bei weit fortgeschrittener Niereninsuffizienz (CKD G5 und CKD G5D) häufig auftritt, sollte eine Knochenbiopsie vor Therapieeinleitung erwogen werden. Das Ergebnis der Knochenbiopsie kann dann Basis einer rationalen Therapiewahl darstellen.

### Therapieindikation – Interventionsschwelle

Aktuell gibt es keine publizierten Daten zur optimalen Interventionsschwelle für die Einleitung einer Osteoporosetherapie bei Patienten mit CKD 4–5. Die nachfolgenden Empfehlungen basieren daher auf der Expertenmeinung der Arbeitsgruppe dieser Leitlinie und orientieren sich an den Empfehlungen für die Allgemeinbevölkerung [74].

#### Sekundärprävention

Bei Auftreten einer osteoporotischen Fraktur (spontan oder nach inadäquatem Trauma) besteht eine manifeste Osteoporose. Bei CKD G4–5 liegen die Wahrscheinlichkeiten für Hospitalisierung, Folgefrakturen, Verlust von Selbstständigkeit im Alltag und auch Mortalität nochmals höher als die ohnehin schon hohen Raten dieser Endpunkte nach osteoporotischen Frakturen in der nierengesunden Allgemeinbevölkerung [75–78]. Eine Osteoporosetherapie sollte daher dem Patienten angeboten werden. Insbesondere nach Oberschenkelhalsfraktur sollte eine Therapieeinleitung erfolgen.

#### Primärprävention

Unter Primärprävention wird in dieser Leitlinie die Vermeidung von Knochenbrüchen bei Patienten ohne vorangegangene Frakturen verstanden. Für die Primärprävention von osteoporotischen Brüchen ist auch für die Allgemeinbevölkerung die Datenlage limitiert, da in den meisten Interventionsstudien die Mehrzahl der Studienteilnehmer eine prävalente Fraktur aufwies und es sich somit bei der Therapie um eine Sekundärprävention handelte. Für die Allgemeinbevölkerung konnte für Alendronat [79] und Denosumab [80] in der Primärprävention gezeigt werden, dass bei erhöhtem Frakturrisiko (identifiziert über eine Knochendichtemessung im osteopenen oder osteoporotischen Bereich) die Frakturraten durch die Behandlung gesenkt werden. Für Patienten mit CKD G4–5 stehen aktuell keine Daten zur Effektivität von Osteoporosetherapien für die Primärprävention zur Verfügung. Die Expertenmeinung der Arbeitsgruppe ist daher, sich an den Empfehlungen für die Allgemeinbevölkerung zu orientieren [74]. Da der FRAX-Algorithmus das wahre Frakturrisiko bei CKD-Patienten wahrscheinlich etwas unterschätzt, entspricht dies einem eher konservativen Therapiezugang. Bei hohem Risiko (FRAX-Score: 10-Jahres-Frakturrisiko > 20 % für eine „major osteoporotic fracture“ [MOF] oder > 5 % für eine Hüftfraktur [HF]) wird empfohlen, eine Osteoporosetherapie mit dem Patienten zu besprechen und ggf. auch einzuleiten.

## Spezifische Therapie der Osteoporose bei CKD

Patienten mit CKD können sowohl erhöhte als auch erniedrigte Knochenumsatzraten aufweisen, wie bereits im CKD-MBD-Kapitel erläutert wurde. Vom pathophysiologischen Gesichtspunkt erscheint es sinnvoll, Patienten mit erhöhtem Knochenstoffwechsel mit antiresorptiven (antikatabolen) Therapien zu behandeln. Umgekehrt könnten Patienten mit niedrigem Knochenstoffwechsel eher von einer osteoanabolen Therapie profitieren. Ob eine antiresorptive Therapie bei CKD-Patienten mit niedrigem Knochenstoffwechsel aber dennoch Vorteile bezüglich Frakturprävention bringen kann, wird aktuell in Expertenkreisen kontroversiell diskutiert [81, 82].

Sämtliche Evidenz bezüglich Senkung des Frakturrisikos durch verschiedene medikamentöse Osteoporosetherapien bei Patienten mit CKD-MBD im Stadium G3–5 stammt aus Post-hoc-Analysen von Phase-III-Studien mit den entsprechenden Einschränkungen bezüglich ihrer Aussagekraft. Die Tab. 4 gibt eine Übersicht über die verfügbare Osteoporosetherapie im Kontext der CKD. Für alle Therapien gilt, dass der Kalzium- und Vitamin-D-Status vor Beginn einer Osteoporosetherapie überprüft und bei Mangel substituiert werden sollten, um eine klinisch relevante Hypokalziämie zu vermeiden. Ein unkontrollierter Hyperparathyreoidismus sollte ebenfalls vor der Einleitung einer Osteoporosetherapie ausreichend behandelt sein. Antiresorptive Medikamente, insbesondere Denosumab als sehr potentes Antiresorptivum, können bei fortgeschrittener CKD eine iatrogene Hypokalzämie induzieren [83]. Eine Kontrolle des Serumkalziums sollte daher kurzfristig (1 bis 2 Wochen) nach Beginn einer antiresorptiven Therapie erfolgen.

**Table 4:** 

Medikament	Renale Elimination	Wirksamkeit bei mittelgradiger CKD (post hoc bei postmenopausalen Frauen)	Klinische Studien bei fortgeschrittener CKD	Sicherheit/Risiken	Kommentare
Bisphosphonate (Alendronat [84, 85], Ibandronat [86], Risedronat [87, 88], Zoledronat^a^)	Ja	Frakturen ↓	KMD ↑	*Hypokalzämie* (insbesondere i.v.) Ösophagitis (p.o.) Nephrotoxizität (i.v.) Atypische Frakturen, Kieferknochennekrose	Euvolämie Infusionsgeschwindigkeit GFR-Grenzen
Denosumab [83, 89, 90]	Nein	Frakturen ↓	KMD ↑	*Hypokalzämie* Atypische Frakturen, Kieferknochennekrose	Rebound-Effekt nach Absetzen
Raloxifen [91, 92]	Nein	Frakturen ↓	KMD ↑	Venöse Thrombembolien Hitzewallungen	Risikoreduktion für Brustkrebs Frakturreduktion nur für vertebrale Frakturen gezeigt
Hormonersatztherapie (Östrogen ± Gestagen)	Nein	Keine Daten	Keine Daten	Mammakarzinom, venöse Thrombembolien, ischämischer Insult	Unklares Nutzen-Risiko-Verhältnis bei CKD
Teriparatid [93–96]	Nein	Frakturen ↓	KMD ↑	Blutdrucksenkung	Therapiedauer maximal 24 Monate, antiresorptive Anschlusstherapie
Romosozumab [97–99]	Unwahrscheinlich	Keine Daten	KMD ↑	*Hypokalziämie* Kardiovaskuläres Risiko bei CKD 4–5 unklar	Therapiedauer maximal 12 Monate; antiresorptive Anschlusstherapie

Je nach Studie lag der Bereich für „mittelgradige“ CKD bei einer eGFR (bzw. Kreatinin-Clearance) > 30 bis > 45 ml/min/1,73 m^2^ und für „fortgeschrittene CKD“ bei < 45 ml/min/1,73 m^2^ bis zum Dialysestadium

^a^Keine Analyse bezüglich Effektivität bei CKD publiziert

In Anlehnung an internationale Leitlinien [10, 73] wird für die Behandlung der Osteoporose bei Patienten mit CKD empfohlen:

- Falls eine Hypokalziämie vorliegt, sollte diese vor Beginn einer medikamentösen Osteoporosetherapie ausgeglichen werden.
- Bei Patienten mit CKD G1–G2 mit Osteoporose und/oder hohem Frakturrisiko gemäß FRAX (MOF > 20 %, HF > 5 %) wird ein therapeutisches Management wie für die Allgemeinbevölkerung empfohlen.
- Bei Patienten mit CKD G3–G5D und laborchemischen Zeichen einer CKD-MBD sollte diese wie oben angeführt unter entsprechender Laborkontrolle behandelt werden.
- Bei Patienten mit CKD G3b mit PTH im Normbereich und Osteoporose und/oder hohem Frakturrisiko gemäß FRAX (MOF > 20 %, HF > 5 %) wird ebenso eine Behandlung wie für die Allgemeinbevölkerung empfohlen.
- Bei Patienten mit CKD G4–5 und Fragilitätsfrakturen (Sekundärprävention) sollte eine Osteoporosetherapie eingeleitet werden.
- Bei Patienten mit CKD G4–5 mit hohem Frakturrisiko gemäß FRAX (MOF > 20 %, HF > 5 %) sollte eine Osteoporosetherapie erwogen und ggf. auch eingeleitet werden.
- Nach Einleitung einer antiresorptiven Therapie bei Patienten mit CKD G4–5 sollte innerhalb der ersten 1 bis 2 Wochen eine Kalziumkontrolle erfolgen.

### Bisphosphonate

Bisphosphonate (in Österreich verfügbar: Alendronat, Risedronat, Ibandronat, Zoledronat) sind potente Inhibitoren der Knochenresorption. Sie werden an metabolisch aktiven Umbaueinheiten im Knochen abgelagert und bewirken eine Apoptose von Osteoklasten. Die Resorptionsaktivität wird im Gesamtskelett deutlich gedämpft und das Frakturrisiko reduziert.

Oral werden Bisphosphonate nur in geringem Ausmaß (maximal 3 %) resorbiert; die Einnahme erfolgt stets nüchtern in ausreichendem Abstand zur Nahrungsaufnahme, mit ausreichend Wasser und in aufrechter Körperhaltung, um Irritationen der Ösophagusschleimhaut zu vermeiden.

Bei intravenöser Bisphosphonat-Gabe kann, überwiegend bei erstmaliger Verabreichung, eine sog. „Akutphase-Reaktion“ – im Wesentlichen ein grippales Zustandsbild mit Fieber und Muskelschmerzen – auftreten, die in der Regel innerhalb von 36 h nach intravenöser Gabe beginnt und 24–48 h anhält. Eine symptomatische Therapie mit z. B. Paracetamol wird hier allgemein empfohlen.

Bei allen Bisphosphonaten stellen eine Hypokalzämie, eine Gravidität oder eine fortgeschrittene CKD eine Kontraindikation dar.

Da Bisphosphonate renal eliminiert werden, besteht ein gewisses Potenzial für eine direkte Nephrotoxizität. Fälle von akutem Nierenversagen nach i.v.-Bisphosphonat-Gabe wurden berichtet. Es dürfte sich jedoch insgesamt um eine Nebenwirkung handeln, die vor allem i.v.-Bisphosphonate betrifft und selten ist [100]. So wurden i.v.-Bisphosphonate auch in kleineren Studien bei nierentransplantierten Patienten verabreicht, ohne dass eine Nephrotoxizität beobachtet worden wäre [101, 102]. Um das Risiko einer akuten Nephrotoxizität dennoch zu minimieren, sollten insbesondere i.v.-Bisphosphonate nur bei Euvolämie verabreicht werden. Bei Exsikkose sollte die Bisphosphonat-Gabe postponiert werden. Eine langsamere Verabreichung (z. B. Verdopplung der Infusionsdauer) von i.v.-Bisphosphonaten bei Patienten mit CKD kann erwogen werden, um die pharmakologischen Spitzenspiegel zu senken und so möglicherweise das Risiko einer akuten Nierenschädigung weiter zu reduzieren [103]. Aufgrund der renalen Elimination von Bisphosphonaten wurde auch von manchen Klinikern vorgeschlagen, die verabreichte Dosis von i.v.-Bisphosphonaten der GFR anzupassen (Dosisreduktion bei CKD) [103]. Es ist allerdings unklar, ob eine Dosisreduktion von i.v.-Bisphosphonaten bei CKD-Patienten einen Vorteil bezüglich Sicherheit bringt und ob bei reduzierter Dosis eine ausreichende Wirksamkeit noch gegeben ist. Daher empfehlen die Autoren dieser Leitlinie, die regulären Dosierempfehlungen und Nierenfunktionsgrenzen der i.v.-Bisphosphonate gemäß Fachinformationen einzuhalten (Tab. 5) und i.v.-Bisphosphonate nur bei klinischer Euvolämie und mit langsamerer (halber) Infusionsgeschwindigkeit bei Patienten mit CKD zu verabreichen. Bei fortgeschrittener Niereninsuffizienz im Stadium CKD G4 (eGFR 15–29 ml/min/1,73 m^2^) sollten aufgrund der oben genannten Überlegungen präferenziell orale und nicht i.v.-Bisphosphonate zum Einsatz kommen. Auch orale Bisphosphonate sind bei Patienten im Stadium CKD G4 laut aktuellen Fachinformationen (Tab. 5) kontraindiziert (Risedronat) bzw. „nicht empfohlen“ (Alendronat), es handelt sich daher bei Patienten im Stadium CKD G4 um eine Off-label-Behandlung. Die Autoren dieser Leitlinie empfehlen daher, vor Therapiestart mit einem Bisphosphonat im Stadium CKD G4 die Patienten über diesen Umstand aufzuklären und dies auch entsprechend zu dokumentieren. Aufgrund der antiresorptiven Wirkungsweise können bei fortgeschrittener Niereninsuffizienz nach Bisphosphonat-Beginn Hypokalzämien auftreten. Eine Laborkontrolle 1 bis 2 Wochen nach Therapiestart wird daher empfohlen. Bei Patienten mit CKD G4 und unklaren Konstellationen sollte eine Überweisung an eine nephrologische und/oder osteologische Ambulanz bezüglich näherer Evaluierung und Therapieentscheidung erwogen werden. Bei Patienten im Stadium CKD 5D (Dialyse) sollte die Therapie direkt mit dem behandelnden Nephrologen akkordiert werden.

**Table 5:** 

Wirkstoff	Dosis	Kontraindikation
Alendronat	70 mg p.o. 1‑mal/Woche	CrCl < 35 ml/min „Nicht empfohlen“
Ibandronat	3 mg i.v. alle 3 Monate	CrCl < 30 ml/min
Ibandronat	150 mg p.o. 1‑mal/Monat	CrCl < 30 ml/min „Nicht empfohlen“
Risedronat	35 mg p.o. 1‑mal/Woche	CrCl < 30 ml/min
Zoledronat	5 mg i.v. 1‑mal/Jahr	CrCl < 35 ml/min

*CrCl* Creatinine-Clearance, *i.v.* intravenös, *p.o.* per os

Bisphosphonate haben eine lange Verweildauer im Knochen. Residuale Wirkungen auf den Knochenstoffwechsel lassen sich auch nach Beendigung der Bisphosphonat-Therapie nachweisen. Das Auftreten von atypischen Femurfrakturen ist sehr selten, scheint aber unter einer Langzeitgabe mit Bisphosphonaten zuzunehmen. Kiefernekrosen sind bei dieser für Osteoporose zugelassenen Therapie eine sehr seltene Nebenwirkung. Eine Kontrolle des Zahnstatus ist allerdings vor Therapiebeginn empfehlenswert.

Es gibt keine durch Frakturdaten validierten individuellen Entscheidungskriterien für die Wiederaufnahme einer Therapie nach einer Therapiepause oder einen weiteren Therapieverzicht in Abhängigkeit von Veränderungen der KMD, der Knochenumbaumarker oder anderer messtechnischer oder klinischer Kriterien. Datenbankanalysen geben allerdings Hinweise auf einen Wiederanstieg des Knochenbruchrisikos nach Absetzen einer Bisphosphonat-Therapie [74].

### Denosumab

Denosumab ist ein monoklonaler Antikörper gegen RANKL, der die Reifung und Aktivierung der Osteoklasten hemmt. Er wird alle 6 Monate subkutan verabreicht und wird nicht renal eliminiert.

Bei der Behandlung der postmenopausalen Osteoporose ist eine Reduktion von vertebralen und nichtvertebralen Frakturen inklusive proximaler Femurfrakturen in Studien bis zu 10 Jahre nachgewiesen. Die Wirkung ist unabhängig von einer eventuellen Vorbehandlung mit Bisphosphonaten [104]. Die optimale Behandlungsdauer ist nicht definiert. Nach Absetzen von Denosumab kommt es im Gegensatz zu den Bisphosphonaten zu einem raschen Anstieg des Knochenumbaus und in weiterer Folge zu einer Abnahme der Knochenmineraldichte und einer Zunahme der Frakturrate [105]. Nach Beendigung einer Denosumab-Therapie sollte daher eine Bisphosphonat-Therapie angeschlossen werden, um diesen überschießenden Knochendichteverlust abzufangen [105]. Kiefernekrosen und atypische Femurfrakturen sind bei dieser für die Osteoporose zugelassenen Therapie und Dosierung eine sehr seltene Nebenwirkung [74].

Laut aktueller Fachinformation besteht für Denosumab keine Kontraindikation bei eingeschränkter Nierenfunktion, auch nicht im Dialysestadium. Mit abnehmender GFR steigt allerdings die Wahrscheinlichkeit für klinisch relevante schwere Hypokalzämien [83], sodass hier entsprechende Laborkontrollen des Serumkalziums 1 bis 2 Wochen nach Therapiebeginn empfohlen werden. Auch hier sollte bei Patienten im Stadium CKD 5D (Dialyse) die Therapie direkt mit dem behandelnden Nephrologen akkordiert werden.

### Hormonersatztherapie

Vor allem aus Analysen der sog. Women’s Health Initiative(WHI)-Studie gibt es Hinweise, dass eine Behandlung mittels Hormonersatztherapie (englisch: „hormone replacement therapy“ [HRT]), bestehend aus Östrogen mit oder ohne Gestagen, bei postmenopausalen Frauen zu einer verminderten Frakturrate führt [106, 107]. In Subgruppenanalysen bei Frauen unter 60 Jahren erscheint dieser frakturprotektive Effekt einer HRT zumindest fraglich [108]. Post-hoc-Analysen der WHI-Studie zur Frakturprävention einer HRT bei Frauen mit CKD liegen nicht vor.

Die in der WHI verwendete HRT, bestehend aus konjugiertem equinem Östrogen in Kombination mit Medroxyprogesteron-Acetat, wird aktuell nicht mehr verwendet. Für alternative, aktuell verfügbare HRT-Präparate gibt es keine ähnlich zur WHI-Studie gelagerte Evidenz bezüglich Frakturprävention.

Mögliche Nebenwirkungen einer HRT sind eine erhöhte Inzidenz an Mammakarzinomen, venösen Thromboembolien und ischämischen Insulten [106, 107]. Da es sich bei CKD-Patienten um ein kardiovaskuläres Hochrisikokollektiv handelt [109], ist hier das Nutzen-Risiko-Verhältnis einer HRT bezüglich Frakturprävention zumindest unklar und möglicherweise ungünstig.

### Raloxifen

Raloxifen ist zugelassen für die Prävention und Therapie der Osteoporose bei postmenopausalen Frauen. Raloxifen ist ein selektiver Östrogenrezeptormodulator (SERM), der die Knochenresorption hemmt und das Frakturrisiko für vertebrale Frakturen reduziert. Für nichtvertebrale Frakturen und proximale Femurfrakturen liegen keine Frakturpräventionsdaten vor.

Ein bedeutender zusätzlicher Effekt ist die Reduktion des relativen Risikos eines invasiven (Östrogenrezeptor-positiven) Mammakarzinoms um 79 %. Eine unerwünschte Nebenwirkung ist die Erhöhung des thromboembolischen Risikos [74]. Raloxifen ist laut Fachinformation „bei schwerer Nierenfunktionsstörung“ kontraindiziert. In der Fachinformation ist die „schwere Nierenfunktionsstörung“ nicht näher definiert, im Allgemeinen kann darunter ein CKD-Stadium 4 (eGFR < 30 ml/min/1,73 m^2^) verstanden werden.

### Teriparatid

Teriparatid, ein aminoterminales Fragment des Parathormons, wird 1‑mal täglich subkutan über bis zu 24 Monate angewandt. Der osteoanabole Effekt beruht auf einer Beschleunigung der Reifung und Stimulierung von Osteoblasten. Im Anschluss an die anabole Reaktion des Knochens kommt es nach Beendigung der Teriparatid-Therapie wiederum zu einem gesteigerten Knochenabbau, weshalb eine unmittelbare Anschlussbehandlung mit einem Antiresorptivum (Bisphosphonat, Denosumab, SERM) unbedingt notwendig ist [74].

Laut aktueller Fachinformation ist Teriparatid bei „schwerer Nierenfunktionsstörung“ kontraindiziert. Teriparatid ist per se nicht nephrotoxisch, die Kontraindikation stützt sich vermutlich eher auf die mangelnde Datenlage bei schwerer Niereninsuffizienz bei den Zulassungsstudien. In Fallserien konnte bei Dialysepatienten eine Zunahme der Knochendichte unter Teriparatid beobachtet werden [94–96, 110, 111]. Aufgrund von pathophysiologischen Überlegungen erscheint eine Teriparatid-Therapie bei ausgeprägtem Hyperparathyreoidismus nicht sinnvoll, da hier von einem PTH-Überschuss in Kombination mit einer mehr oder weniger ausgeprägten PTH-Resistenz des Knochens ausgegangen wird. Eine zusätzliche PTH-Gabe (bzw. Teriparatid als PTH-Fragment) bringt daher voraussichtlich keinen weiteren therapeutischen Nutzen. Unter Teriparatid kommt es kurzfristig (innerhalb von Stunden) zu einer transienten Hyperkalzämie und dann sekundär zu einer Hyperkalziurie [112], welche potenziell bei Nierenkranken und insbesondere Patienten mit Nephrolithiasis nachteilig sein könnte. Bei Patienten im Stadium CKD G4–5D ist eine Therapie mit Teriparatid eine Off-label-Anwendung. Eine Teriparatid-Therapie erscheint den Autoren dieser Leitlinie bei Patienten im Stadium CKD G4–5D mit niedrigem PTH bzw. gut kontrolliertem sekundären HPT (s. Tab. 3) eine rationale Therapieoption.

### Romosozumab

Romosozumab, ein Anti-Sclerostin-Antikörper, wirkt sowohl osteoanabol als auch antiresorptiv (dualer Wirkmechanismus). Studiendaten bei postmenopausalen Frauen mit einem erhöhten Knochenbruchrisiko zeigen eine außergewöhnlich starke Zunahme der KMD und Reduktion der Frakturrate bei monatlicher Applikation für 12 Monate [113–115]. Romosozumab ist aktuell kontraindiziert bei Patienten mit kardiovaskulären Ereignissen in der Anamnese, womit aufgrund der hohen Prävalenz von kardiovaskulären Erkrankungen bei CKD diese Therapie für viele CKD-Patienten ausscheidet. Zukünftig könnte Romosozumab jedoch eine neue Behandlungsoption auch für manche CKD-Patienten mit Osteoporose darstellen [73]. Präliminäre Daten zeigen einen weitgehend erhaltenen Effekt von Romosozumab auf die Knochendichtezunahmen bei Patienten mit CKD G3 im Vergleich zu Nierengesunden, ohne Auffälligkeiten bezüglich der Sicherheit [97]. Bei CKD G4–5D liegen aktuell nur begrenzte Daten vor. Bei CKD G4–5D kommt es nach Romosozumab-Gabe zu einem deutlichen Abfall des Serumkalziums, dem auch teilweise therapeutisch entgegengesteuert werden musste [98, 99]. Die Anwendung von Romosozumab bei CKD 4–5D wird daher aktuell nur mit großer Vorsicht und engmaschiger Kontrolle empfohlen.

## Physikalisch-rehabilitative Maßnahmen

Nephrologische Patienten können neben der Osteopenie bzw. Osteoporose zahlreiche weitere Krankheitsfolgen aufweisen wie Malnutrition, Sarkopenie, Frailty, urämische Myopathie und Neuropathie, chronische Entzündung, Fatigue, Inaktivität sowie Komorbiditäten (Diabetes, koronare Herzerkrankung, Arthrose und kognitiven Abbau). Diese Folgeerscheinungen bewirken beispielsweise Dekonditionierung, erhöhtes Sturzrisiko und die Unfähigkeit, Aktivitäten des täglichen Lebens (ATLs) selbstständig durchzuführen. Des Weiteren steht Inaktivität in engem Zusammenhang mit raschem Verlust der GFR und exzessiver Mortalität [116, 117], deshalb gilt es, die oben genannten Folgeerscheinungen rehabilitativ zu beeinflussen. Die Empfehlungen zu Krafttraining, Ausdauertraining sowie Koordinationstraining/Sturzprophylaxe sind in Tab. 6 zusammengefasst.

**Table 6:** 

Gesellschaft	Kraft	Ausdauer	Koordination/Sturzprophylaxe
ESSA [125]	2‑mal/Woche, 8 bis 12 Übungen, 10–15 WH, 60–70 % des 1WHM	180 min/Woche, 55–90 % der maximalen HF (Borg 11–16)	10 min, 5‑ bis 7‑mal/Woche
EUROD [73]	Regelmäßig, entsprechend den Fähigkeiten und Bedürfnissen	–	Sturzrisiko regelmäßig untersuchen, ggf. therapieren
WHO [134]	Mindestens 2‑mal/Woche, alle größeren Muskelgruppen, moderate bis höhere Intensität	150–300 min/Woche bei moderater Intensität, oder 75–150 min/Woche bei hoher Intensität, oder Kombination	3‑mal/Woche

Die WHO-Empfehlungen gehen nicht explizit auf CKD-Patienten ein, sondern richten sich an alle Erwachsenen inklusive Personen mit chronischen Krankheiten

*ESSA* Exercise & Sports Science Australia, *EUROD* European Renal Osteodystrophy, *WHO* World Health Organization, *WH* Wiederholungen, *WHM* Wiederholungsmaximum, *HF* Herzfrequenz

### Krafttraining

Krafttraining steigert die Muskelmasse und wirkt osteoanabol [118, 119]. Bei CKD-Patienten [120–122] wird zwar nicht explizit eine direkte osteoanabole Wirkung, jedoch eine Verbesserung der Kraft und Muskelmasse durch Krafttraining beschrieben. Zusätzlich scheint es positiv auf Sturz- und Frakturrisiko zu wirken [123].

### Ausdauertraining

Aerobes Ausdauertraining verbessert die kardiorespiratorische Fitness und Belastbarkeit und senkt den Ruheblutdruck und Puls [124, 125]. Des Weiteren gibt es Hinweise auf eine Steigerung der kardialen Auswurffraktion, eine bessere glykämische Kontrolle, eine Verbesserung psychosozialer Parameter [117] sowie eine Stabilisierung der GFR und Reduktion der Mortalität [126].

Einige Arbeiten berichten auch von einer möglichen positiven Wirkung durch aerobes Ausdauertraining auf Knochenstoffwechselparameter [127, 128]. Des Weiteren könnte aerobes Training den altersbedingten Verlust an Knochenmasse verlangsamen, eine eindeutige positive Wirkung auf die Knochendichte wurde jedoch nicht berichtet [129, 130]. Aerobes Training kann das Sturzrisiko reduzieren, wenn auch geringer als kombiniertes Kraft- und Ausdauertraining [131]. Außerdem scheint regelmäßiges aerobes Training, welches in höherer aerober Fitness resultiert, die Erholung nach intensiven Aktivitäten zu beschleunigen [132].

### Koordinationstraining/Sturzprophylaxe

Viele Fragilitätsfrakturen, insbesondere Oberschenkelhalsfrakturen, geschehen im Rahmen von Sturzereignissen im Alltag. Ein Sturzprophylaxetraining sowie die Beseitigung von sturzfördernden Faktoren im Umfeld sind daher eine sinnvolle Maßnahme zur Prävention von osteoporotischen Frakturen.

### Beweglichkeitstraining

Beweglichkeitstraining ist sowohl in der Prävention (vermindertes Sturzrisiko [133]) als auch in der Rehabilitation nach bereits erfolgter Fraktur günstig. Bei relevanten Einschränkungen der Aktivitäten des täglichen Lebens sind ergotherapeutische Interventionen sinnvoll [116]. Ernährungsberatung/-therapie wird für CKD-Patienten ebenso empfohlen [1, 126].

### Rehabilitation nach Frakturen

Erleiden CKD Patienten eine Fraktur, ist es wichtig, die orthopädisch-traumatologischen Aspekte mit den oben erwähnten generellen physikalisch-rehabilitativen Maßnahmen zu kombinieren.

#### Wirbelkörperfraktur

Bei osteoporotischen Wirbelkörperfrakturen sollten eine multimodale Schmerztherapie (medikamentös und physikalisch), ggf. eine Versorgung mit funktionellen Orthesen (meist für 8 bis 12 Wochen) sowie ein rasches Wiedererlangen der Mobilität erfolgen, um Komplikationen der Immobilität wie Pneumonie oder Dekonditionierung zu verhindern [135].

Im Rahmen der Bewegungstherapie erfolgen in der Frühphase neben der Remobilisierung auch Instruktionen bezüglich Ergonomie (z. B. En-bloc-Drehen), isometrische Stabilisierungsübungen zur Verhinderung einer Kyphosierung sowie ggf. ein stufenweiser Abbau des Mieders [136]. Des Weiteren sollte ein Training der Sensomotorik erfolgen, um das Risiko für weitere Stürze zu reduzieren, sowie die Beseitigung von Stolperfallen und das Absetzen von Sturz-begünstigenden Medikamenten [136]. Funktionelle Orthesen können Schmerzen lindern, die Rückenmuskulatur aktivieren und die Aktivitäten des täglichen Lebens erleichtern [135].

Erfahrungsgemäß kommt es bei osteoporotischen Wirbelkörperfrakturen gemäß radiologischen Kriterien nach etwa 3 bis 4 Monaten zur Ausheilung [137]. Dies kann bei fortschreitender Niereninsuffizienz jedoch auch länger dauern. Jedenfalls soll ab der radiologisch bestätigten Heilung auch an der Beweglichkeit der Wirbelsäule gearbeitet werden.

Kommt es aufgrund der Fraktur zu funktionellen Einschränkungen, sollten eine ergonomische Beratung inklusive Evaluation einer Hilfsmittelversorgung sowie ein Training bezüglich Aktivitäten des täglichen Lebens erfolgen, um die Selbstständigkeit wiederzuerlangen. Bei schwerwiegenden Einschränkungen sind unter Umständen eine pflegerische Unterstützung und/oder psychosoziale Intervention erforderlich [136].

Eine individualisierte und supervidierte Trainingstherapie kann in Abhängigkeit der klinischen Beschwerden und des radiologischen Heilungsverlaufs vorsichtig ab 4 bis 6 Wochen nach einer osteoporotischen Wirbelkörperfraktur begonnen werden [138, 139]. In der Akutphase (erste 3 Monate) soll der Fokus auf die Aktivierung und Verbesserung der Ausdauer der Rückenstrecker sowie auf ein Balancetraining gelegt werden. Zusätzlich können beispielsweise die Kniestrecker und/oder Kniebeuger ohne fortgeleitete Belastung der Wirbelsäule (z. B. mittels „leg extension“ bzw. „leg curl“) trainiert werden. In der subakuten Phase (ab ca. 3 Monaten bzw. ab erfolgter Frakturheilung) soll das Balancetraining intensiviert und um funktionelles Training bzw. Krafttraining ergänzt werden [138].

Supervidiertes Krafttraining bei postmenopausalen Frauen [140] und Männern [141] mit sehr niedriger Knochendichte scheint sicher zu sein. Insbesondere wurde in diesen Studien von einer Verbesserung von Kyphosen berichtet, es traten keine neuen Frakturen auf, und es kam auch zu keinem Progress prävalenter Frakturen.

#### Schenkelhalsfraktur

Die Schenkelhalsfraktur ist meist ein Zeichen für komplexe Funktionsstörungen. Deshalb sind die rehabilitativen Ziele vielfältig, und es sollten medikamentöse, diätetische, bewegungstherapeutische und ergonomische Maßnahmen inklusive einer Hilfsmittelversorgung sowie physikalische Modalitäten kombiniert werden [142, 143].

Eine Atemtherapie zur Pneumonieprophylaxe sollte frühzeitig zum Einsatz kommen, unserer Meinung nach bereits präoperativ. Die Remobilisierung soll rasch erfolgen (s. Tab. 7), bereits ab dem ersten postoperativen Tag und mindestens 1‑mal täglich [144]. Abhängig von der Operationsmethode variieren die Belastbarkeit sowie die freigegebenen Bewegungsumfänge. Die Bewegungstherapie gestaltet sich außerdem entsprechend den Phasen der Bindegewebsheilung [143, 145]. In der Akutphase (Tage 0 bis 5) stehen Atemtherapie, entstauende Maßnahmen, passiv/assistive Gelenkmobilisation sowie Stehversuche und Gangschulung im Vordergrund, wohingegen in der Proliferationsphase (Tage 5 bis 21) an Koordinations- und Sensomotoriktraining sowie der weiteren Verbesserung der Beweglichkeit und des Ganges gearbeitet wird. In der Konsolidierungsphase (Tage 21 bis 60) erfolgen v. a. Narbentherapie, Kräftigung und Sturzprophylaxe. In der Umbauphase liegt der Schwerpunkt auf der medizinischen Trainingstherapie.

**Table 7:** 

Wirbelkörperfraktur	Schenkelhalsfraktur
Multimodale Schmerztherapie (medikamentös, physikalisch)	
Ggf. Miederversorgung	Atemtherapie (bereits präoperativ)
Rasche Remobilisierung	
Ergonomie	Belastbarkeit/Bewegungsumfang je nach Operationsmethode
Isometrische Übungen	Akutphase: Atemtherapie, Entstauung, Mobilisation, Stehversuche
Sturzprophylaxe	Proliferationsphase: Mobilisierung, Koordination, Gangschulung
Ggf. funktionelle Orthesen	Konsolidierungsphase: Narbentherapie, Sturzprophylaxe, Kräftigung, Elektrostimulation
Beweglichkeit (ab ca. 3 bis 4 Monaten), Krafttraining (allgemein ab ca. 6 Monaten, spezifisch ggf. bereits ab ca. 6 Wochen)	Umbauphase: med. Trainingstherapie
Aktivitäten des täglichen Lebens, Hilfsmittel	

### Praxisanleitung

In Tab. 8 finden sich praktische Beispiele sowohl für die Prophylaxe als auch für die Therapie.

**Table 8:** 

Trainingsart	Umsetzung	Beispiele
Krafttraining großer Muskelgruppen	2- bis 3‑mal wöchentlich; 8 Wiederholungen; Widerstand, mit dem 10 Wiederholungen durchgeführt werden können; 2 Sets; 1 min Pause	Kniestrecker, Kniebeuger Gesäß‑, Bauch‑, Rücken‑, Schulterblatt‑, Arm- und Nackenmuskulatur
Ausdauertraining	4‑mal wöchentlich 40 min; Intensität, bei der kurzes Sprechen noch möglich ist	Flottes Gehen, Nordic Walking, Schwimmen, Radfahren, Skilanglauf, lockeres Wandern
Beweglichkeitsübungen	3- bis 7‑mal wöchentlich	Täglich: Radfahren im Liegen, Vorfüße kreisen, Arme kreisen, Schultern kreisen, Kopf drehen und Nicken 3‑mal/Woche Dehnen, 2‑mal 30–60 s halten dazwischen locker lassen: Waden, Hüftstrecker, Hüftbeuger, Adduktoren, Rumpf, Pectoralis, Nacken 3‑mal/Woche Mobilisieren: „Katzenbuckel“ für LWS/BWS, Rotation plus Nicken sowie Seitneigen für HWS, Arme im Liegen über Kopf ablegen für Schultern, aufgestellte Beine im Liegen nach re./li. ablegen, Kopf dreht in die Gegenrichtung, jeweils 5 Wiederholungen
Koordinationstraining/Sturzprophylaxe, insbesondere bei Sturzrisiko	3‑mal wöchentlich	Einbeinstand mit/ohne Anhalten, auf einem Strich gehen, rückwärtsgehen, Stehübungen auf labilen Unterlagen, z. B. Therapiekreisel, Gehen auf Zehenspitzen, Gehen auf Fersen, 30 s bis 2 min pro Übung, 2 bis 5 Wiederholungen Achtung: Während des Trainings besteht ein erhöhtes Risiko für Stürze, entsprechende Vorsichtsmaßnahmen werden empfohlen

Die Adaptierung (Widerstand) definiert sich durch die gewählte Belastung in Kilogramm. Die Frequenz (Einheiten pro Woche, Wiederholungsanzahl …) ist davon unbeeinflusst

Das Übungsniveau (z. B. offene oder geschlossene Augen bei Balancetraining) ist sowohl im präventiven als auch im rehabilitativen Setting individuell anzupassen
